# VGF Peptides in Cerebrospinal Fluid of Patients with Dementia with Lewy Bodies

**DOI:** 10.3390/ijms20194674

**Published:** 2019-09-20

**Authors:** Inger van Steenoven, Barbara Noli, Cristina Cocco, Gian-Luca Ferri, Patrick Oeckl, Markus Otto, Marleen J. A. Koel-Simmelink, Claire Bridel, Wiesje M. van der Flier, Afina W. Lemstra, Charlotte E. Teunissen

**Affiliations:** 1 Alzheimer Center Amsterdam, Department of Neurology, Amsterdam Neuroscience, Vrije Universiteit Amsterdam, Amsterdam UMC, 1081 HV Amsterdam, The Netherlands; wm.vdflier@amsterdamumc.nl (W.M.v.d.F.); a.lemstra@amsterdamumc.nl (A.W.L.); c.teunissen@amsterdamumc.nl (C.E.T.); 2 Neurochemistry Laboratory and Biobank, Department of Clinical Chemistry, Amsterdam Neuroscience, Vrije Universiteit Amsterdam, Amsterdam UMC, 1081 HV Amsterdam, The Netherlands; mja.koel-simmelink@amsterdamumc.nl (M.J.A.K.-S.); c.bridel@vumc.nl (C.B.); 3 NEF-laboratory, Department of Biomedical Sciences, University of Cagliari, 09402 Monserrato, Italy; barbaranoli@yahoo.it (B.N.); cristina.cocco@unica.it (C.C.); ferri@unica.it (G.-L.F.); 4 Department of Neurology, Ulm University Hospital, 89081 Ulm, Germany; patrick.oeckl@uni-ulm.de (P.O.); markus.otto@uni-ulm.de (M.O.); 5 Department of Epidemiology and Biostatistics, Amsterdam Neuroscience, Vrije Universiteit Amsterdam, Amsterdam UMC, 1081 HV Amsterdam, The Netherlands

**Keywords:** VGF, cerebrospinal fluid, dementia with Lewy bodies, synaptic dysfunction

## Abstract

In a previous proteomic study, we identified the neurosecretory protein VGF (VGF) as a potential biomarker for dementia with Lewy bodies (DLB). Here, we extended the study of VGF by comparing levels in cerebrospinal fluid (CSF) from 44 DLB patients, 20 Alzheimer’s disease (AD) patients, and 22 cognitively normal controls selected from the Amsterdam Dementia Cohort. CSF was analyzed using two orthogonal analytical methods: (1) In-house-developed quantitative ELISA and (2) selected reaction monitoring (SRM). We further addressed associations of VGF with other CSF biomarkers and cognition. VGF levels were lower in CSF from patients with DLB compared to either AD patients or controls. VGF was positively correlated with CSF tau and α-synuclein (0.55 < *r* < 0.75), but not with Aβ1-42. In DLB patients, low VGF levels were related to a more advanced cognitive decline at time of first presentation, whereas high levels of VGF were associated with steeper subsequent longitudinal cognitive decline. Hence, CSF VGF levels were lower in DLB compared to both AD and controls across different analytical methods. The strong associations with cognitive decline further points out VGF as a possible disease stage or prognostic marker for DLB.

## 1. Introduction

Dementia with Lewy bodies (DLB) is a common cause of dementia in the elderly, accounting for up to 20% of dementia cases [1]. Next to cognitive decline, core features of DLB include parkinsonism (rigidity, bradykinesia, and postural instability), visual hallucinations, fluctuations in cognition and attention, and rapid eye movement (REM) sleep behavior disorder (RBD) [2]. The neuropathological hallmark of DLB is the accumulation of α-synuclein aggregates in Lewy bodies and Lewy neurites throughout the brain [3,4]. In addition, synaptic dysfunction is also a central feature [5,6,7]. Diagnosis of DLB during life is challenging, largely due to a highly variable clinical manifestation and overlap in signs, symptoms and pathology with both Alzheimer’s disease (AD) and Parkinson’s disease (PD). Cerebrospinal fluid (CSF) biomarkers have been proven useful in AD, where a typical CSF profile of decreased levels of amyloid-β 1-42 (Aβ1-42), combined with increased levels of total and phosphorylated tau (t-tau, p-tau) protein levels, supports the diagnosis of AD [8]. Reliable CSF biomarkers to support the diagnosis of DLB are still lacking.

In a recent study done by our group, the secretory granule protein VGF (non-acronymic) was identified as a novel potential biomarker candidate for DLB in cerebrospinal fluid (CSF) using an in-depth proteomics workflow. In fact, VGF was decreased in CSF from patients with DLB compared to cognitively normal (age-matched) controls. VGF is a secreted neuronal and endocrine precursor protein whose expression is induced by brain-derived neurotrophic factor (BDNF) [9,10]. VGF is processed into a number of bioactive peptides which have been implicated in the regulation of energy homeostasis and metabolism as well as neurogenesis and synaptic plasticity [9,10]. In cultured neurons and animal models, VGF-derived peptides regulate synaptic plasticity in the hippocampus, pointing to a stimulatory role for VGF in memory formation [11,12]. In addition, full-length VGF functions in the regulated secretory pathway, modulating the formation of large dense-core vesicles (LDCVs) [13]. VGF-derived peptides have recently received growing attention because of their role in learning and memory [11,12] and potential role in the pathophysiology of psychiatric and neurodegenerative disorders [14]. For example, the VGF-derived peptide GGEE-45 (VGF373-417) has been shown to be decreased in CSF from in Alzheimer’s disease (AD) patients [15]. The excitatory actions of VGF on synaptic plasticity, its role in biogenesis of synaptic vesicles, and its described role in pathophysiology of neurodegenerative diseases that involve synaptic dysfunction, all point to a potential role for VGF in synaptic (dys)function.

The main aim of the present study was to extend the clinical validation of CSF VGF in a relatively large cohort consisting of patients with DLB, compared to either AD and cognitively normal controls. We used two different analytical methods: (1) In-house-developed quantitative competitive enzyme linked immunosorbent assays (ELISA), and (2) selected reaction monitoring (SRM). In addition, we aimed to explore possible correlations between VGF and other CSF biomarkers, as well as to investigate whether VGF might have a role in predicting cognitive decline and survival in DLB patients.

## 2. Results

### 2.1. Patient Characteristics

Demographic and clinical characteristics of the diagnostic groups are presented in Table 1. There were no group differences in sex, education, or apolipoprotein (APOE) genotype, while age was slightly higher in DLB patients compared to controls. As expected, patients with dementia had lower Mini-Mental State Examination (MMSE) scores compared to controls (AD vs. controls: *p* < 0.001; DLB vs. controls: *p* < 0.05). Similarly, patients with dementia had lower cognitive domain scores compared to controls (*p* < 0.05). CSF Aβ1-42 was lower, and tau and p-tau levels were higher, in patients with AD (*p* < 0.001), whereas their levels in patients with DLB were in-between those of patients with AD and controls (*p* < 0.05). There were no significant differences in CSF α-synuclein levels between DLB patients and controls (*p* > 0.05).

### 2.2. CSF VGF Levels in DLB, AD, and Controls

**Figure 1:**
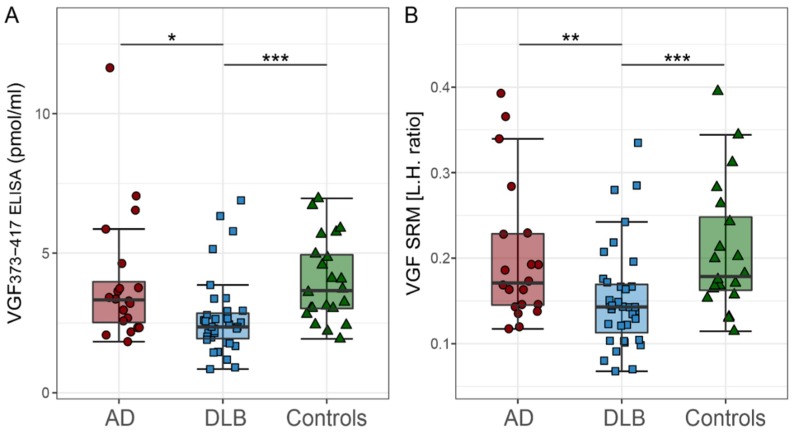
CSF VGF in DLB, AD and controls. (**A**) CSF levels of VGF373-417 measured with ELISA and (**B**) VGF measured with SRM. The line through the middle of each box corresponds to the median and the lower and the upper lines to the 25th and 75th percentile, respectively. The whiskers extend from the 5th percentile on the bottom to the 95th percentile on the top. Differences between groups were assessed with GLM corrected for age and sex. AD = Alzheimer’s disease; DLB = dementia with Lewy bodies; GLM = general linear model; SRM = selected reaction monitoring; VGF = neurosecretory protein VGF. * *p* < 0.05, ** *p* < 0.01, *** *p* < 0.001.

Levels of VGF373-417, as measured by ELISA, were lower in DLB patients than in either AD patient, or in controls (*p* < 0.05 and *p* < 0.001, respectively; Table 1 and Figure 1A). Similarly, levels of VGF in CSF, as assessed by SRM, were lower in DLB patients compared with either AD patients or with controls (*p* < 0.01 and *p* < 0.001, respectively; Table 1 and Figure 1B). CSF VGF levels did not differ significantly between AD patients and controls (*p* > 0.05).

Both the VGF373-417 and VGF485-503 ELISA assays yielded broadly comparable results. Further comparative analyses were focused on VGF373-417 peptide. VGF485-503 data are depicted in the Supplementary Materials.

### 2.3. Correlations Between VGF and Other CSF Biomarkers

We performed Spearman correlation analysis to assess associations between the CSF VGF levels and other CSF biomarker levels throughout all investigated groups (Supplementary Table S1). VGF373-417 ELISA correlated highly with VGF SRM (*r* > 0.834, *p* < 0.001). In DLB and controls, but not in AD, VGF373-417 and VGF SRM correlated positively with CSF tau, p-tau, and α-synuclein (0.552 < *r* < 0.914, *p* < 0.001). No associations between VGF and Aβ1-42 were found among patients with DLB and AD. In controls, however, VGF levels correlated positively with CSF Aβ1-42 (*r* > 0.509, *p* < 0.05).

### 2.4. VGF and Cognitive Decline in DLB Patients

**Figure 2:**
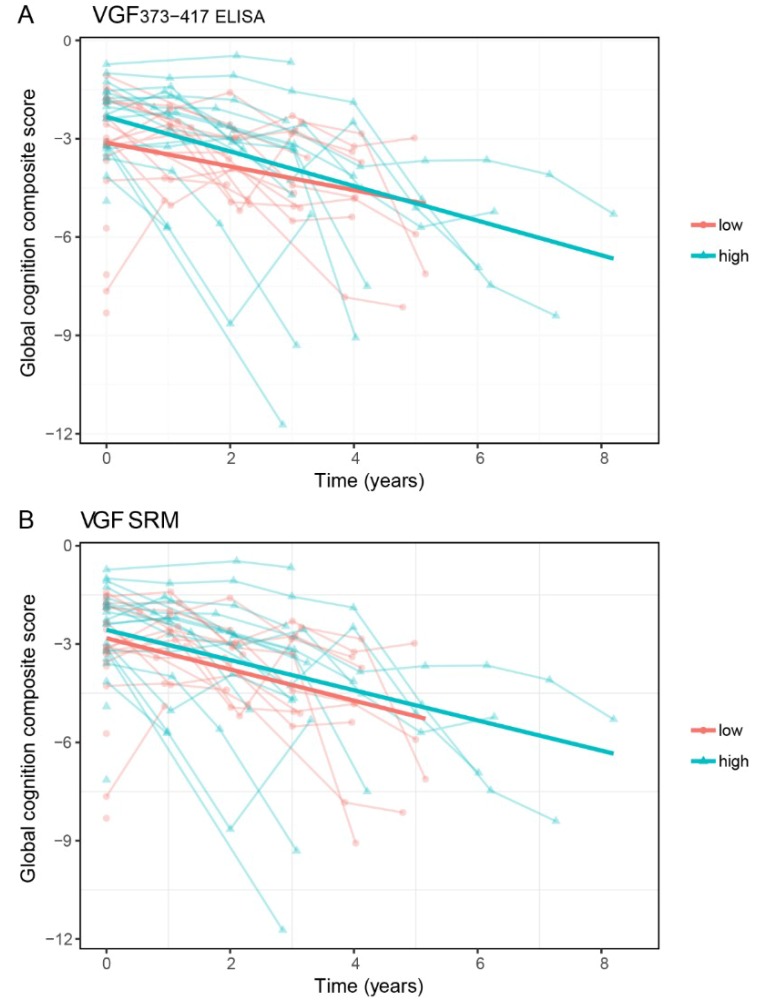
CSF VGF levels and global cognitive score in DLB. (**A**) Associations between baseline CSF levels of VGF373-417 levels measured with ELISA and subsequent global cognition in the DLB patient group (*n* = 44), (**B**) VGF measured with SRM. Associations are shown using linear regression lines, with all DLB patients classified into two groups with a split-half approach using the median value as cut-off point, according to their CSF VGF levels. For the linear mixed model statistical analysis, continuous CSF VGF levels were used.

Subsequently, we used age-, sex-, and education-adjusted linear mixed models to assess the associations between CSF VGF and cognitive decline. We first investigated associations between CSF VGF levels and MMSE scores. VGF373-417 and VGF SRM showed a distinct association with MMSE, both at patients’ first assessment at the memory clinic and over follow-up (Table 2). Next, we assessed associations between CSF VGF levels and cognitive domain scores and a global cognition composite score at the time of patients’ first assessment at the memory clinic. In line with the associations, we found with MMSE, DLB patients with lower CSF VGF (below median) showed lower scores in the attention domain, executive function domain, and language domain, compared to DLB patients with higher VGF (above median). In addition, lower CSF VGF levels were also associated with poor global cognition scores (Table 2, Figure 2). In contrast, linear mixed models showed that higher VGF levels (as above) were associated with an ensuing steeper rate of decline in executive functioning, language performance, and global cognition during follow-up (Table 2, Figure 2). CSF VGF measures were not related to decline in memory performance or visuospatial functioning. Results of individual patients’ cognitive tests are presented in Supplementary Table S2.

### 2.5. VGF and Survival in DLB Patients

Finally, to assess the association between CSF VGF and survival, we performed Cox proportional hazards models adjusted for sex and age. During follow-up, 25 DLB patients (56%) died and 19 DLB patients were alive (median survival time: 5.32 years (95% CI: 4.7–7.1 years)). Cox proportional hazard models revealed that CSF VGF levels peptides were not associated with mortality (VGF373-417: HR = 0.78 [0.5–1.2], *p* = 0.26; VGF SRM: HR = 0.79 [0.5–1.2], *p* = 0.27).

## 3. Discussion

In this study, we successfully extended and validated the findings of our previous CSF proteomics study in DLB using two different analytical methods in a well-characterized and independent cohort that included patients with DLB, AD and cognitively normal controls. We showed that CSF levels of VGF measured with both ELISA and SRM were robustly lower in patients with DLB compared to both AD patients and controls. In addition, our results suggest that in DLB low VGF levels were associated with worse cognition at baseline, whereas relatively high levels were associated with a subsequent more rapid cognitive deterioration in DLB.

VGF is a secretory granule protein and peptide precursor [10] and has been proposed as a marker for synaptic dysfunction. Growing evidence indicates that synaptic dysfunction, and as a consequence, neurotransmitter deprivation, is involved in the pathophysiology of DLB [5,6,7,16]. In general, changes in synaptic function are reflected by alterations in the levels of synaptic proteins [17]. For example, cortical reductions in different presynaptic and postsynaptic proteins have been observed in DLB [16]. In line with this, we observed lower levels of VGF in CSF of patients with DLB using two different analytical methods. Clinical and preclinical studies link VGF-derived peptides to other neurodegenerative diseases including amyotrophic lateral sclerosis (ALS) [18,19,20,21], frontotemporal dementia (FTD) [22], Parkinson’s disease (PD) [23] and AD [15,23,24,25,26,27,28,29,30,31,32]. More specific, previous studies have demonstrated a reduction in VGF-derived peptides in the CSF of patients affected by ALS (VGF398-411), FTD (VGF26-62), and AD (e.g., TLQP-62, VGF373-417, VGF64-80, VGF268-278) compared to control subjects [15,18,19,22,24,25,26,27,28,29,30,31,32]. Although previous work has not yet examined the levels of VGF in the CSF in PD and other α-synucleinopathies, a decrease of TPGH and NERP-1 peptides was revealed in the parietal cortex of PD patients [23]. Such findings support the hypothesis that synaptic dysfunction may be a nonspecific proxy for decline in multiple neurodegenerative diseases [16]. In contrast with this hypothesis and with previous studies, we found no difference in VGF levels in CSF between our AD patients and controls, using both ELISA and SRM methods. The source of this apparent discrepancy is not clear. Even though we note that crude VGF levels were visually lower in AD compared to controls, these differences did not reach statistical significance. This might be (partly) due to the relatively small group of AD patients used for this study and the large variance within the AD group. Another possible explanation could be that that different VGF-derived peptides may differ in their response and change in different conditions, highlighting the need of further studies to evaluate the role of different VGF-derived peptides in the pathogenesis of neurodegenerative diseases.

The exact pathophysiological mechanisms behind the decreased VGF levels in CSF and synaptic dysfunction in DLB is not clear. Growing evidence indicates that neocortical synaptic dysfunction in DLB is probably caused, at least in part, by presynaptic deposition of pathological proteins, such as toxic α-synuclein oligomers leading to alterations in levels of other synaptic proteins and functional disconnection [5,6,7,33]. Downregulation of the proteins RAB3A and SNAP-25 [34] and synaptopodin [35] in DLB suggests defective synaptic transmission in DLB. VGF is similarly suggested to be involved in synaptic transmission [10]. We reported positive correlations between VGF, tau, p-tau, and α-synuclein levels in DLB patients, suggesting a common underlying mechanism. These findings are consistent with previous studies in AD patients, showing clearly positive correlations between CSF VGF and tau, p-tau [36,37], and support the notion that CSF concentrations of VGF and possibly other synaptic proteins are directly or indirectly associated with the severity of synaptic dysfunction.

Synaptic dysfunction is thought to precede neuronal degeneration, and may correlate more directly with cognitive decline than pathological hallmarks such as Lewy body pathology. Our finding that DLB patients with low CSF VGF levels had lower cognitive performance scores at first visit to the memory clinic, whereas DLB patients with initial high levels of VGF showed steeper cognitive decline over time, might suggest a dynamic pathophysiological trajectory with progression of the disease. This hypothesis is supported by a recent study in patients across the disease stages of AD showing that CSF levels of multiple synaptic proteins, including VGF, were increased in patients with mild cognitive impairment (MCI) due to AD compared to controls subjects and patients with AD dementia [36]. In addition, longitudinal measurements of VGF in CSF of patients affected with AD showing that VGF levels decrease with time as the disease progresses [25]. We could speculate that a compensatory mechanism (e.g., reorganization of presynaptic end terminal [38,39,40]) in patients in an early disease stage is accompanied by an upregulation of synaptic protein levels, whereas levels of these proteins declines in later disease stages as a result of further established synaptic dysfunction, associated with a simultaneously or subsequent cognitive decline. This needs further investigation in longitudinal studies with extensive clinical follow-up and repeated lumbar punctures including a large number of DLB patients across various stages of the disease.

Among the strengths of our study are the relatively large number of DLB patients for this type of experiment and the use of two different analytical methods. The fact that both analytical methods showed essentially similar results confirms the robustness of our findings. Furthermore, etiological diagnosis was either supported by biomarkers or confirmed by autopsy. In addition, control subjects included in the present study had normal CSF AD biomarker levels at first presentation and persevered normal cognitive function for at least two years. Using these inclusion criteria, we could almost certainly rule out AD in these individuals with subjective cognitive decline (SCD). We therefore consider it unlikely that our results are significantly biased by clinical misdiagnosis. Some limitations of the present study also need to be mentioned. First, we focused here on two peptides derived from the VGF protein only, rather than including a broader comparison with other VGF peptides. In view of pathophysiological implication of different VGF peptides which are endowed with differing bioactivity and roles [9,10], the results reported should be expanded to further studies evaluating other VGF peptides, for example TLPQ- and NERP-peptides. The different VGF peptides may differ in their response and change in different conditions, hence it would be relevant to evaluate the involvement of VGF peptides in other neurodegenerative conditions. VGF data on patients with other neurodegenerative diseases, including PD, ALS, and FTD, were not available for the present study. Thus, it is not possible at this time to incorporate the present results into the broader range of neurodegenerative diseases. Finally, although clinical follow-up extended up to nine years for individual patients, it was only 2.6 years on average. Moreover, clinical follow-up duration in DLB patients with low VGF levels was shorter (mean: 2.1–2.3 years) compared to DLB patients with high VGF levels (mean: 2.8–3.0 years). This difference in follow-up duration could potentially have biased our findings. However, we observed no difference in survival time between DLB patients with low VGF levels versus high VGF levels, suggesting that the DLB patients with low VGF levels were not lost to follow-up due to a faster and more severe disease course.

## 4. Materials and Methods

### 4.1. Study Population

We included 44 patients with DLB, 20 patients with AD, and 22 individuals with subjective cognitive decline (SCD) who served as controls. The above-mentioned patients and controls were selected from the Amsterdam Dementia Cohort [41], consisting of patients who were assessed at the memory clinic of the Alzheimer Center Amsterdam between January 2000 and December 2017.

All selected patients and controls underwent an extensive standardized and multidisciplinary work-up, including medical history, physical and neurological examination, neuropsychological testing, electroencephalography (EEG), brain magnetic resonance imaging (MRI), and laboratory testing, including lumbar puncture. Diagnoses were made in a multidisciplinary consensus meeting.

DLB was diagnosed according to the international diagnostic consensus criteria for probable DLB [2]. The diagnosis of DLB was supported by (123)I-FP-CIT-SPECT (DAT-SPECT) scan showing presynaptic dopaminergic deficits (*n* = 41, 93%) or was confirmed at autopsy (*n* = 3, 7%). Clinical diagnosis of AD was established according to National Institute on Aging and Alzheimer’s Association (NIA-AA) criteria. All AD patients had positive AD CSF biomarkers (CSF Aβ1-42, total tau and p-tau). Individuals were labeled as SCD when they presented at the memory clinic with subjective cognitive complaints, but (i) ancillary investigations were normal, and (ii) criteria for Mild Cognitive Impairment (MCI), dementia, or other neurological or psychiatric disorder(s) known to cause cognitive complaints were not met (i.e., cognitively normal elderly). In addition, the individuals with SCD we selected showed negative AD CSF biomarkers, as well as their cognitive function on neuropsychological testing remained normal for at least two years at follow-up after first presentation at the memory clinic.

The study was approved by the local medical ethics committee and all subjects gave their written informed consent for the use of their clinical data and CSF for research purposes.

### 4.2. Cogntive Assessment

A standardized neuropsychological test battery was used to measure performance on five cognitive domains. The memory domain included the total immediate and delayed recall of the Dutch version of Rey Auditory Verbal Learning Test and the total recall on condition A of the Visual Association Test. The attention domain consisted of Digit Span Forward, Trail Making Test (TMT) part A and the Stroop word and color tasks. To measure executive functioning, we used TMT part B, color-word task of the Stroop test, Digits Span Backward, and Letter Fluency (sum of letters D, A & T). The language domain included Category Fluency test (animals, 1 min) and VAT naming condition. The visuospatial domain was assessed using Dot Counting and Number Location of the Visual Object and Space Perception battery (VOSP). In addition, we used Mini-Mental State Examination (MMSE) as an indication of global cognitive performance. Raw test scores for TMT and Troop were inverted as higher scores indicate worse performance. To obtain cognitive domain scores, all raw test scores were converted to z-scores using the mean and standard deviation of equivalent neuropsychological test scores from an independent reference group of healthy controls (*n* = 533, age = 59.7 ± 9.8 years, 46% male, MMSE = 28.9 ± 1.0). Next, we created composite cognitive domain scores by averaging z-scores for the corresponding tests per domain. Composite scores were only calculated if there were data available on ≥2 tests within that specific domain, otherwise that domain score was classified as missing. In addition, a global cognitive composite score that combined all 16 neuropsychological test scores across five different cognitive domains was calculated.

Follow-up for all patients took place by annual routine visits to the memory clinic, in which physical and neurological examinations and cognitive assessment were repeated. Each DLB patient underwent at least one neuropsychological examination. Follow-up cognitive data were available in 33 (75%) DLB patients, with a mean follow-up time of 2.7 ± 2.2 years. Follow-up extended up to nine years for individual patients.

### 4.3. Mortality

For each DLB patient, all-cause mortality information (died yes/no, and date of death) was updated using the Dutch municipal population register (date searched: 30 December 2018). Causes of death were not indicated in this register. We defined survival time as the time between (i) the date of the patient’s first visit at the memory clinic, and (ii) either the date of death or the above date (30 December 2018) for alive patients.

### 4.4. CSF Procedures

In line with international biobanking consensus guidelines [42], CSF was obtained by lumbar puncture between the L3/L4, L4/L5, or L5/S1 intervertebral space using a 25-gauge needle and a syringe, collected into 10 mL polypropylene tubes (Starstedt, Nümbrecht, Germany), centrifuged at 1800× *g* at 4 °C for 10 min, and aliquoted in polypropylene tubes of 0.5 mL. Part of the CSF was used for routine analyses, including leukocyte and erythrocyte count, glucose concentration, total protein concentration and levels of Aβ1-42, total tau and p-tau were measured with commercially ELISA assays (Innotest^®^, Fujirebio, Gent, Belgium). Previously validated reference values for AD based on the biobank data were used: <813 pg/mL for Aβ1-42, >375 pg/mL for tau and >52 pg/mL for p-tau [43,44]. The ratio of CSF total tau and Aβ1-42 was used to determine the presence of an AD profile in CSF (CSF tau/Aβ1-42 ≥ 0.52, [45]. The remaining CSF was processed similarly, but stored directly in aliquots of 0.5 mL at −80 °C until analysis.

CSF samples assessed in the present study were taken at the patient’s first presentation at the memory clinic.

### 4.5. Alpha-Synuclein Analysis

CSF levels of α-synuclein were assessed with an in-house validated immunoassay from ADx Neurosciences (Gent, Belgium) [46].

### 4.6. VGF Analysis

#### 4.6.1. ELISA Analysis

The natural peptides (a) VGF485-503 or NAPP-19 (so named on the basis of its N-terminal four amino acid residues, and its length) and (b) VGF373-417 or GGEE-45 (as above) were identified in human CSF, and/or in the TT human thyroid cell line, respectively [15,47]. Synthetic peptides corresponding to the N-terminal portion of either natural peptide (human VGF485-493 and human VGF373-382) were conjugated to keyhole limpet haemocyanin (KLH) via an additional Cysteine residue at their C-terminus, and used for rabbit immunizations [10,23]. Competitive assays were set up for (a) human VGF485-503 and (b) human VGF373-417, respectively, as described [48,49,50]. Briefly, plates were coated with the corresponding peptide in carbonate/bicarbonate buffer (pH 9.6), blocked in PBS-Tween 20 (0.01 mol/L PO_4_, pH 7.2–7.4, 0.15 mol/L NaCl, 0.5 g/L Tween 20) containing normal donkey serum (90 mL/L), aprotinin (20 nmol/L) and EDTA (1 g/L), and incubated (3 h, at room temperature) with a mixture of primary antibody (in the same medium) and serial dilutions of either standard peptide, or sample/s. After primary incubation, plates were washed, hence treated with biotinylated secondary antibody (Jackson, West Grove, PA, USA), streptavidin-peroxidase conjugate (Biospa, Milan, Italy), and tetramethylbenzidine (X-tra Kem-En-Tec, Taastrup, Denmank). The reaction was stopped with HCl (1 mol/L), and optical density was measured at 450 nm using a multilabel plate reader (Chameleon: Hidex, Turku, Finland). Synthetic peptides corresponding to the full natural peptides (“a” and “b”, above) were used as standards. Assay characterization showed: 50% inhibition of signal with 1 nmol/L and 10 pmol/L standard peptide; intra- and inter-assay variation (CV%) of 3% and 11%, 4% and 10%; deviation from parallelism in serial sample dilutions <15% and <10% for the VGF485-503 and VGF373-417 assay, respectively. Recovery of standard peptide added to human CSF was >80% for both assays. To gain some insight as to assay specificity for N-terminally cleaved peptides, versus the same sequence within N-terminally extended forms, or the VGF precursor, synthetic peptides were used containing an additional N-terminal amino acid residue (see basic amino acid sites immediately preceding the natural peptides “a” and “b”, above, within VGF, and most likely implicated in their N-terminal cleavage: Human VGF Lys481-Arg482-Lys483-Lys484, and human VGF Arg371-Arg372, respectively). Cross-reactivity with such N-terminally extended peptides was 0.1% and 0.5% in the VGF485-503 and VGF373-417 assay, respectively, hence indicating a high specificity of either assay for the relevant cleaved peptide.

The results reported mostly focus on the VGF373-417 peptide. Analysis of the VGF485-503 peptide yielded comparable findings, depicted in Supplementary Figure S1.

#### 4.6.2. SRM Analysis

For SRM analysis, 200 µL of CSF was spiked with Triethylammonium bicarbonate (TEAB) buffer and a quantitative protein epitope signature tag (QPrEST), kindly provided by Atlas Antibodies AB, #QPrEST20926) of VGF as internal standard. Samples were reduced and alkylated with 1 mM (Tris(2-carboxyethyl)phosphine hydrochloride) (TCEP) and 1 mM 2-chloroacetamide (CAA) at 95 °C for 10min. Proteins were digested for 16 h at 37 °C by adding 1.2 µg trypsin/LysC (Promega). Digestion was stopped by addition of 800 µL 1.25% trifluoroacetic acid (TFA) and peptides were transferred to strong cation exchange STAGE-Tips [51] by centrifugation. Peptides were washed with 0.2% TFA, followed by 75 mM ammonium acetate/20% acetonitrile/0.5% formic acid, and eluted with 125 mM ammonium acetate/20% acetonitrile/0.5% formic acid. After vacuum drying, peptides were dissolved in 30 µL 6% acetonitrile/0.1% TFA and analyzed by LC-SRM. Analysis of VGF was performed with a QTRAP6500 mass spectrometer (AB Sciex), Eksigent MicroLC200, and Agilent 1260 HPLC pump. Peptides were loaded on a C18 PepMap100, 5 µm, 0.3 × 5 mm trap column (Thermo Fisher Scientific). Separation was performed on an Eksigent HALO Fused-core C18, 2.7 µm, 0.5 × 100 mm column at 40 °C with mobile phase A: 4% DMSO/0.1% formic acid, and mobile phase B: 4% DMSO/96% acetonitrile/0.1% formic acid and a linear gradient from 1–30% B within 9.85 min. The following transitions of the proteotypic VGF peptide AQEEAEAEER (aa586-595) were measured: 581.3–962.4 (y8), 581.3–833.4 (y7), 581.3-704.3 (y6) (light peptide); 586.3–972.4 (y8), 586.3–843.4 (y7), 586.3–714.3 (y6) (heavy peptide). For relative quantification, the light-to-heavy (L/H) peptide ratio (mean of the three transitions) was calculated using Skyline v4.2. CSF QC samples were included in each run. Intra-assay CVs were 5.1–7.9%.

### 4.7. Statistical Analyses

Statistical analyses were performed in R v.3.5.1 ‘Feather Spray’. We assessed differences in patient characteristics using univariate analysis of variance (ANOVA) with post-hoc False Discovery Rate (FDR) correction, χ^2^ test, or Kruskal Wallis H test variance with post hoc FDR correction when appropriate. Differences in CSF biomarkers were assessed using general linear model (GLM) corrected for age and sex. CSF tau, p-tau, and VGF were logarithmically transformed before the analyses because of skewed values. We assessed associations of VGF with the established CSF AD biomarkers, Aβ1-42, tau and p-tau, and α-synuclein using Spearman’s correlations. FDR corrections were used to adjust *p*-values for multiple comparisons. Associations between VGF and cognition at baseline and longitudinal changes over time were assessed with linear mixed models adjusted for age, sex, and education. The models included terms for time (years), VGF levels and an interaction term of VGF level*time as independent variables and MMSE scores, cognitive domain z-scores and global cognition composite score as dependent variables. For all models, a random intercept was assumed. VGF levels were transformed to z-scores. A Beta-coefficient of one (β = 1) therefore implies that one standard deviation increase in VGF levels was associated with one standard deviation increase in cognition score. For illustrative purposes, we dichotomized VGF levels (DLB patients were classified into two groups with a split-half approach using the median value of VGF as cut-off point). Finally, we tested the effect of CSF VGF levels on time to death. Cox proportional hazards models with age and sex as covariates were used to calculate hazards ratios for determining the effect of CSF VGF levels on time to death. Statistical significance was set at *p* < 0.05 for main effects and *p* < 0.10 for interaction effects.

## 5. Conclusions

In conclusion, the present study represents a first step in validating novel synaptic biomarkers for DLB, by showing that VGF levels are lower in patients with DLB compared to both AD patients and cognitively normal controls across different analytical methods. Our results highlight the importance of synaptic dysfunction in DLB. The strong association with cognitive decline might indicate that VGF is useful as a disease stage or prognostic marker in an early symptomatic stage of the disease. Further investigation of synaptic pathophysiology in the clinical continuum of DLB is likely to provide new insight.

## Figures and Tables

**Table 1:** Demographics, clinical characteristics, and CSF biomarker levels according to diagnosis.

	DLB (*n* = 44)	AD (*n* = 20)	Controls (*n* = 22)
**Demographics**			
Female (*n*, %)	5 (11%)	2 (10%)	4 (18%)
Age	67 (6) ^a^	65 (6)	63 (5)
Education, years	10 [9–13]	10 [9.5–13]	13 [10–13]
MMSE	23 [21–26] ^b,c^	18 [16–22] ^b^	29 [27–30]
APOEε4 carrier (*n*, %)	23 (54%)	12 (63%)	7 (32%)
**Cognitive function z-scores**			
Memory	−2.67 (1.67) ^b^	−3.12 (1.91) ^b^	−0.26 (0.75)
Attention	−2.89 (2.18) ^b,c^	−4.39 (3.41) ^b^	−0.27 (0.71)
Executive functions	−4.32 (2.79) ^b^	−5.27 (3.21) ^b^	−0.41 (0.88)
Language	−1.13 (0.69) ^a,c^	−2.44 (2.71) ^b^	−0.20 (0.56)
Visual spatial functions	−0.85 (0.94) ^d^	−2.37 (1.68) ^b^	−0.38 (0.96)
Global cognition score	−2.90 (1.69) ^b,d^	−4.41 (2.32) ^b^	−0.32 (0.62)
**CSF AD biomarkers**			
Aβ1-42 (pg/mL)	780 [658–977] ^b,d^	586 [492–642] ^b^	1040 [913–1150]
tau (pg/mL)	292 [224–367] ^a,d^	596 [498–905] ^b^	194 [169–256]
p-tau (pg/mL)	47 [35–59] ^a,d^	87 [66–122] ^b^	39 [30–46]
CSF α-synuclein (pg/mL)	1805 [1540–2169]	NA	1466 [1280–1911]
**CSF VGF**			
VGF373-417 ELISA (pmol/mL)	2.5 [2.1–3.4] ^b,c^	3.3 [2.5–3.9]	3.6 [3.0–4.9]
VGF SRM [L.H.ratio] ^§^	0.14 [0.12–0.18] ^b,c^	0.17 [0.14–0.22]	0.17 [0.16–0.24]

Data are presented as mean (SD), median [interquartile range] or *n* (%). Differences in patient characteristics were assessed with ANOVA, χ^2^, and Kruskal Wallis H tests were performed where appropriate. Differences in CSF biomarker levels were assessed with GLM corrected for age. CSF tau, p-tau, α-synuclein and VGF were logarithmically transformed for the analyses because of skewed values, but are presented here as raw data. ^§^ DLB, *n* = 44; AD, *n* = 20; controls, *n* = 21. Cognitive function z-scores were calculated using the mean and SD of an independent normal reference group. Abbreviations: Aβ1-42 = amyloid β1-42; AD = Alzheimer’s disease; APOE = apolipoprotein; CSF = cerebrospinal fluid, DLB = dementia with Lewy bodies; ELISA = enzyme linked immunosorbent assay; MMSE = Mini-Mental State Examination; p-tau = tau phosphorylated at threonine 181; SRM = selected reaction monitoring; VGF = neurosecretory protein VGF. ^a^: *p* < 0.05 compared to controls; ^b^: *p* < 0.001 compared to controls; ^c^: *p* < 0.05 compared to AD; ^d^: *p* < 0.001 compared to AD.

**Table 2:** Effects of CSF VGF on change in cognitive performance over time in DLB.

Cognitive Domains	Estimated Baseline Performance		Estimated Change Over time	
	β (SE)	*p*	β (SE)	*p*
**VGF373-417 ELISA**				
MMSE	1.26 (0.56)	0.03 *	−0.32 (0.14)	0.02 *
Memory	0.00 (0.24)	0.99	0.04 (0.06)	0.52
Attention	0.87 (0.42)	0.03 *	−0.29 (0.12)	0.01 *
Executive functions	1.27 (0.43)	0.004 **	−0.31 (0.10)	0.002 **
Language	0.21 (0.09)	0.03 *	−0.04 (0.02)	0.10
Visuospatial functions	0.46 (0.29)	0.12	−0.01 (0.09)	0.91
Global cognition	0.69 (0.25)	0.008 **	−0.16 (0.06)	0.008 **
**VGF SRM**				
MMSE	1.11 (0.56)	0.04 *	−0.38 (0.13)	0.006 **
Memory	0.04 (0.23)	0.85	0.00 (0.06)	0.97
Attention	0.92 (0.41)	0.02 *	−0.26 (0.12)	0.03 *
Executive functions	1.32 (0.42)	0.003 **	−0.35 (0.10)	<0.001 ***
Language	0.19 (0.09)	0.05	−0.03 (0.02)	0.25
Visuospatial functions	0.53 (0.29)	0.07	−0.09 (0.09)	0.31
Global cognition	0.72 (0.25)	0.006 **	−0.18 (0.05)	0.002 **

Data are presented as standardized β (SE). The models included terms for time, the biomarker under investigation and biomarker*time interaction and sex, age and education. For all models, a random intercept and fixed slope were assumed. CSF VGF levels were log-transformed ad transformed to z-scores prior to analysis. β’s for biomarkers represent the estimated change in z-score for each standard deviated increase in biomarker level at baseline, while β’ for the biomarker*time interaction represent estimated change in z-score for each year of follow-up. * *p* < 0.05, ** *p* < 0.01, *** *p* < 0.001.

## Supplementary Materials

Supplementary materials can be found at https://www.mdpi.com/1422-0067/20/19/4674/s1.

## Abbreviations

Aβ_1-42_	amyloid-β 1-42
AD	Alzheimer’s disease
APOE	Apolipoprotein
CSF	cerebrospinal fluid
DAT-SPECT	^123^I[FP-CIT] single photon emission computed tomography
DLB	dementia with Lewy bodies
EEG	electroencephalography
ELISA	enzyme-linked immunosorbent assays
MCI	mild cognitive impairment
MMSE	Mini-Mental State Examination
MRI	magnetic resonance imaging
p-tau	tau phosphorylated at threonine 181
RBD	REM (rapid eye movement) sleep behavior disorder
SCD	Subjective cognitive decline
SRM	selected reaction monitoring
t-tau	total tau
VGF	neurosecretory protein VGF
