# Supplementary material for: VGF Peptides in Cerebrospinal Fluid of Patients with Dementia with Lewy Bodies
Source: Int J Mol Sci. 2019 Sep 20;20(19):4674. doi: 10.3390/ijms20194674 (PMC6801397; doi:10.3390/ijms20194674)
Supplement: Supplementary file 1 [file ijms-20-04674-s001.pdf]

Supplementary Materials

VGF Peptides in cerebrospinal fluid of patients with dementia with Lewy Bodies

Inger van Steenoven 1,2,*, Barbara Noli 3, Cristina Cocco 3, Gian-Luca Ferri 3, Patrick Oeckl 4, Markus Otto 4, Marleen J.A. Koel-Simmelink 2, Claire Bridel 2, Wiesje M. van der Flier 1,5, Afina W. Lemstra 1 and Charlotte E. Teunissen 1,2

[1] Alzheimer Center Amsterdam, Department of Neurology, Amsterdam Neuroscience, Vrije Universiteit Amsterdam, Amsterdam UMC, Amsterdam, The Netherlands
2 Neurochemistry Laboratory and Biobank, Department of Clinical Chemistry, Amsterdam Neuroscience, Vrije Universiteit Amsterdam, Amsterdam UMC, Amsterdam, The Netherlands
3 NEF-laboratory, Department of Biomedical Sciences, University of Cagliari, Monserrato, Italy
4 Department of Neurology, Ulm University Hospital, Ulm, Germany
5 Department of Epidemiology and Biostatistics, Amsterdam Neuroscience, Vrije Universiteit Amsterdam, Amsterdam UMC, Amsterdam, The Netherlands
* Correspondence: i.vansteenoven@amsterdamumc.nl; Tel.: +31-20-4440685

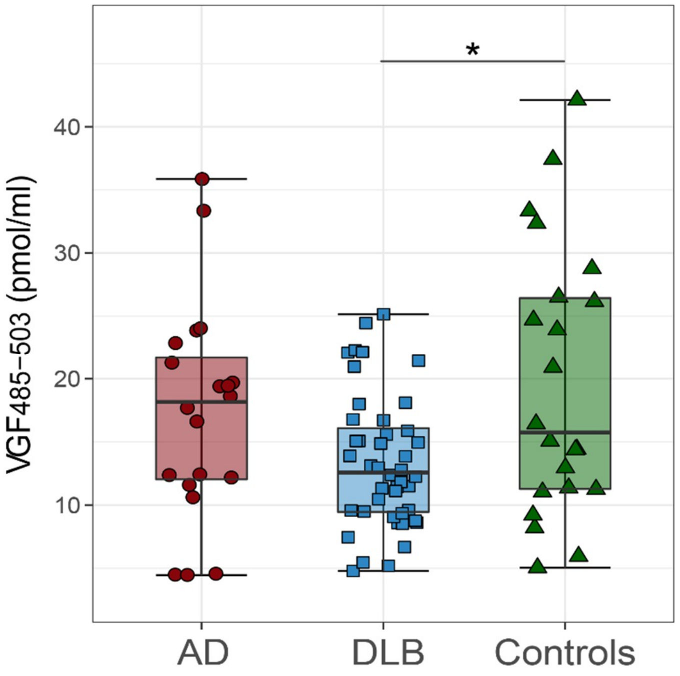

Figure S1. CSF $VGF_{485-503}$ levels in DLB, AD and controls
The line through the middle of the boxed corresponds to the median and the lower and the upper lines to the 25th and 75th percentile, respectively. The whiskers extend from the 5th percentile on the bottom to the 95th percentile on the top. Differences between groups were assessed with GLM corrected for age and sex. AD = Alzheimer's disease; DLB = dementia with Lewy bodies; VGF = Neurosecretory protein VGF.
* $p<0.05$

Table S1. Associations between CSF biomarkers

	**Total group (n=86)**		**DLB (n-44)**		**AD (n=20)**		**Controls (n=22)**	
Protein	$VGF_{373\text{-}417}$ ELISA	VGF SRM	$VGF_{373\text{-}417}$ ELISA	VGF SRM	$VGF_{373\text{-}417}$ ELISA	VGF SRM	$VGF_{373\text{-}417}$ ELISA	VGF SRM
**$VGF_{373\text{-}417}$ ELISA**	-	0.892***	-	0.894***	-	0.908***	-	0.834***
**VGF SRM**	0.892***	-	0.894***	-	0.908***	-	0.834***	-
**$A\beta_{1\text{-}42}$**	0.111	0.024	0.057	-0.111	-0.250	-0.296	0.509*	0.735***
**tau**	0.285**	0.328**	0.551***	0.624***	0.326	0.418	0.787***	0.857***
**p-tau**	0.361***	0.436***	0.563***	0.657***	0.415	0.508*	0.679***	0.778***
**α-synuclein**	0.621***	0.624***	0.731***	0.748***	NA	NA	0.910***	0.914***

Associations were assessed with spearman correlation coefficient. FDR corrections were used to adjust *p* values for multiple comparisons. * *p<0.05*, ** *p<0.01*, *** *p<0.00*

International Journal of
Molecular Sciences

MDPI

Table S2. Effects of CSF VGF on change in cognitive performance over time

	Estimated baseline performance		Estimated change over time	
	β (SE)	p	β (SE)	p
**$VGF_{373\text{-}417}$ ELISA**				
Memory				
RAVLT immediate recall	0.20 (0.11)	0.09	-0.03 (0.02)	0.22
RAVLT delayed recall	0.13 (0.12)	0.28	-0.01 (0.02)	0.46
VAT A	-0.33 (0.63)	0.59	0.12 (0.18)	0.48
Attention				
Digit span forward	-0.06 (0.13)	0.63	0.00 (0.03)	0.96
TMT A	2.68 (0.88)	0.003**	-0.61 (0.24)	0.01*
Stroop 1	0.49 (0.51)	0.33	-0.23 (0.19)	0.22
Stroop 2	0.58 (0.59)	0.32	-0.38 (0.20)	0.05
Executive functions				
Digt span backward	0.01 (0.10)	0.86	0.00 (0.03)	0.99
Stroop 3	4.60 (1.75)	0.01*	-1.32 (0.52)	0.01*
TMT B	1.53 (0.37)	<0.001***	-0.36 (0.10)	<0.001***
Letter fluency	0.14 (0.13)	0.30	-0.02 (0.03)	0.49
FAB	0.42 (0.40)	0.29	-0.08 (0.10)	0.42
Language				
Boston Naming Test	0.21 (0.13)	0.11	-0.08 (0.04)	0.04*
Category Fluency	0.24 (0.12)	0.05	-0.03 (0.03)	0.24
Visuospatial functions				
VOSP number location	0.76 (0.26)	0.005**	-0.06 (0.08)	0.45
VOSP dot counting	0.12 (0.44)	0.77	0.15 (0.14)	0.29
**VGF SRM**				
Memory				
RAVLT immediate recall	0.24 (0.11)	0.03*	-0.05 (0.02)	0.07
RAVLT delayed recall	0.16 (0.12)	0.20	-0.02 (0.02)	0.32
VAT A	-0.33 (0.62)	0.59	0.04 (0.18)	0.82
Attention				
Digit span forward	0.02 (0.01)	0.86	0.00 (0.03)	0.98
TMT A	2.67 (0.86)	0.003**	-0.52 (0.24)	0.03*
Stroop 1	0.72 (0.50	0.15	-0.28 (0.18)	0.13
Stroop 2	0.55 (0.58)	0.34	`-0.27 (0.19)	0.16

	**Estimated baseline performance**		**Estimated change over time**	
	***β* (SE)**	**p**	***β* (SE)**	**p**
Executive functions				
Digt span backward	0.00 (0.10)	0.97	-0.00 (0.02)	0.75
Stroop 3	4.55 (1.72)	0.01*	-1.18 (0.50)	0.02*
TMT B	1.28 (0.37)	0.001**	-0.24 (0.10)	0.011*
Letter fluency	0.17 (0.13)	0.20	-0.00 (0.03)	0.95
FAB	0.54 (0.40)	0.18	-0.10 (0.10)	0.34
Language				
Boston Naming Test	0.17 (0.13)	0.20	-0.06 (0.04)	0.14
Category Fluency	0.23 (0.12)	0.06	-0.04 (0.03)	0.20
Visuospatial functions				
VOSP number location	0.71 (0.26)	0.01*	-0.12 (0.08)	0.16
VOSP dot counting	0.22 (0.44)	0.61	0.06 (0.14)	0.67

Data are presented as standardized *β* (SE). The models included terms for time, the biomarker under investigation and biomarker*time interaction and sex, age and education. For all models a random intercept and fixed slope were assumed. CSF VGF levels were log-transformed ad transformed to z-scores prior to analysis. β's for biomarkers represent the estimated change in z-score for each standard deviated increase in biomarker level at baseline, while β'for the biomarker*time interaction represent estimated change in z-score for each year of follow-up.). Z scores for TMT and Stroop tests were inverted as higher scores indicate worse performance. * *p<0.05*, ** *p<0.01*, *** *p<0.001*.
